# Ampicillin- and Multidrug-Resistant *Escherichia coli* and *Enterococcus* spp. in Costa Rican Wastewater and Surface Water

**DOI:** 10.3390/antibiotics14101024

**Published:** 2025-10-14

**Authors:** Eleanor A. Brodrick, Adriana González-Fernández, Andrew M. Kramer, Valerie J. Harwood

**Affiliations:** 1 Department of Integrative Biology, University of South Florida, Tampa, FL 33620, USA; ebrodrick@usf.edu (E.A.B.); amkramer@usf.edu (A.M.K.); 2 Miami Waterkeeper, Miami, FL 33114, USA

**Keywords:** antibiotic resistance, wastewater treatment, environmental AMR, tropics, pathogens

## Abstract

Antibiotic-resistant bacteria (ARB) such as *Escherichia coli* and *Enterococcus* released into surface waters have strong potential to impact human health. We assessed the prevalence of antibiotic-resistant bacteria (ARB) and multidrug-resistant (MDR) bacteria in undisinfected wastewater effluent in a tropical estuary that receives the discharge from a major wastewater treatment plant (WWTP) in Costa Rica. *Methods:* We quantified culturable ampicillin-resistant (ampR) and (MDR) *E. coli* and *Enterococcus* in wastewater influent from hospital and residential sources, effluent, and estuarine receiving waters of the secondary-treated effluent of a WWTP. AmpR isolates confirmed to species or genus were tested for resistance against six additional antibiotic classes. *Results:* The proportion of ampR *E. coli* (18%) was significantly greater than that of ampR enterococci (4%) but neither differed among sites. AmpR *E. coli* concentrations were significantly different by site (estuary, 3.9 log_10_ CFU/100 mL vs. untreated residential wastewater, 6.4 log_10_ CFU/100 mL), but ampR enterococci concentrations were consistent among sites. MDR *E. coli* and *Enterococcus* were most prevalent in hospital wastewater (57% and 45% of ampR isolates, respectively), but were found at all sites. MDR *E. coli* and *Enterococcus* isolates resistant to five antibiotics were isolated from the estuary, and gentamicin-resistant *Enterococcus* were isolated only from effluent. *Conclusions:* Undisinfected effluent is a source of ARB and MDR opportunistic pathogens in the tropical estuary and has the potential to impact the health of beachgoers. Our findings highlight the importance of rigorous treatment of wastewater effluent, including disinfection, as a contribution to efforts to achieve effective stewardship of antibiotics.

## 1. Introduction

The World Health Organization (WHO) has called antibiotic resistance the greatest global health threat of the 21st century [1]. A systematic analysis of antibiotic resistance estimated 1.27 million deaths worldwide due to antibiotic resistance in 2019 [2]. The continued rise in infections caused by multidrug-resistant (MDR) bacteria (i.e., resistant to at least one antibiotic in three or more antibiotic categories) [3] exacerbates the antibiotic resistance threat, as these infections are more difficult to treat, and more likely to lead to poor outcomes for the patients [4].

Antibiotic resistance has critical consequences for low- and middle- income countries [5,6]. The highest burdens of infections caused by ARB occur in low-resource settings, which have a higher burden of infectious disease due to poverty and insufficient health systems [2,7]. Antibiotic resistance is a national public health problem in Costa Rica. ARB and MDR bacteria have been reported in hospitals and the community throughout the territory and in the livestock sector [8,9]. Clinical data regarding ARB of public health concern in Costa Rica are readily available, while data on ARB isolated from wastewater and the environment are scarce [9]. Furthermore, the regulation of antibiotic use in Costa Rica is less stringent than in more developed countries. For example, Costa Rican law has required a prescription for the sale of antibiotics since 1998, but illegal acquisition of antibiotics is enough of a concern to draw the attention of the local media [10,11,12].

Aquatic ecosystems contain a pool of ARB and antibiotic resistance genes (ARGs) of natural and anthropogenic origin, making them potential reservoirs of antibiotic resistance and habitats where antibiotic resistance could be disseminated [13]. Untreated or inefficiently treated wastewater from wastewater treatment plants (WWTP), particularly those that receive hospital wastewater, have been described as hot spots for the accumulation and spread of ARB and their genes in the environment [14,15,16,17]. The use of reclaimed water for agricultural irrigation, as well as the use of animal manure to fertilize crops, can facilitate the spread of antibiotic resistance through soil [18,19] and into the aquatic environment through coastal runoff [20].

Clinically relevant ARB and associated ARGs can be found in recreational waters and pose a threat to human health [21]. ARB can enter bathers via ingestion [22] or open wounds [23]. Studies on antibiotic-resistant *E. coli* have demonstrated that humans in recreational water bodies run the risk of ingestion of ARB [22,24,25]. Evidence of infection with ARB from recreational waters comes from case studies, including infection with ARB from a French river [26] and from exposure to seawater in China [23]. However, more data are needed to fully understand the concentrations of ARB in recreational water and the risk that these ARB pose to human health [21,22].

Relatively little is known about the prevalence of ARB in wastewater and recreational waters in tropical countries [27]. Most studies have been performed in temperate regions, where environmental conditions (i.e., rainfall) affect microbiological water quality differently [28]. The influence of environmental variables on the distribution of ARGs and a gradient of increased relative abundances of ARGs in middle latitudes compared to high and low latitudes has been previously reported [6]. Antibiotic contaminants in low- and middle-income economies characteristic of many tropical countries are higher when compared to high-income countries [5,6]. Factors such as lower GDP per capita [29], insufficient sanitation infrastructure [5,7], limited access to healthcare [7,29], and antibiotic misuse [29,30] have been invoked to explain the discrepancy. Antibiotic contamination may lead to an increase in ARB because the presence of antibiotics even at low concentrations in vitro can exert a selective pressure on bacterial populations [31].

The fecal indicator bacteria (FIB) *Escherichia coli* and *Enterococcus* spp. are members of the normal flora of the majority of animals, including humans, but they are also opportunistic pathogens [32,33]. FIB are readily cultured and are used to assess microbial water quality in research and regulatory applications [33,34,35]. The WHO Global Antimicrobial Resistance and Use Surveillance System (GLASS), has a module directed towards extended- spectrum *β*-lactamase producing *E. coli* as a key indicator for global surveillance of antibiotic resistance [36]. *E. coli* can readily acquire ARGs by horizontal gene transfer, and it frequently acquires extended-spectrum *β*-lactamases, which are an important cause of multidrug resistance in Gram-negative bacteria worldwide [37,38]. *Enterococcus* spp. are Gram-positive and thus can be used as a counterpoint to *E. coli* for investigating resistance to other clinically important antibiotics [39].

Puntarenas, Costa Rica, is an appropriate test site to explore the prevalence of antibiotic resistance through wastewater treatment stages and the extent to which these bacteria are discharged into the environment. Costa Rica is a tropical middle-income country [40], and meets the parameters for an increased relative abundance of ARGs and antibiotic contaminants. One WWTP receives both hospital and residential wastewater, allowing for comparison between the sources of AMR FIB. The effluent of the WWTP is discharged without disinfection into Puntarenas Estuary, which is adjacent to a popular beach. This enables testing for differences in susceptibility to multiple classes of antibiotics of *E. coli* and *Enterococcus* spp. among these sources. In this study, samples were taken from each of the potential sources, analyzed for total FIB concentrations as well as ampicillin-resistant (ampR) concentrations, and the ampR FIB tested for additional antibiotic resistances.

## 2. Results

Unless intermediate resistance is specified, all references to resistance refer to full antibiotic resistance as specified by CLSI [41]. The term enterococci is used to describe any characteristic colony growth on mEI, a selective-differential media, following US EPA Method 1600 [42], while the term *Enterococcus* is used for any enterococci isolate that has been confirmed to the genus level by qPCR.

### 2.1. Frequency and Concentration of AmpR E. coli and Enterococci

**Figure 1:**
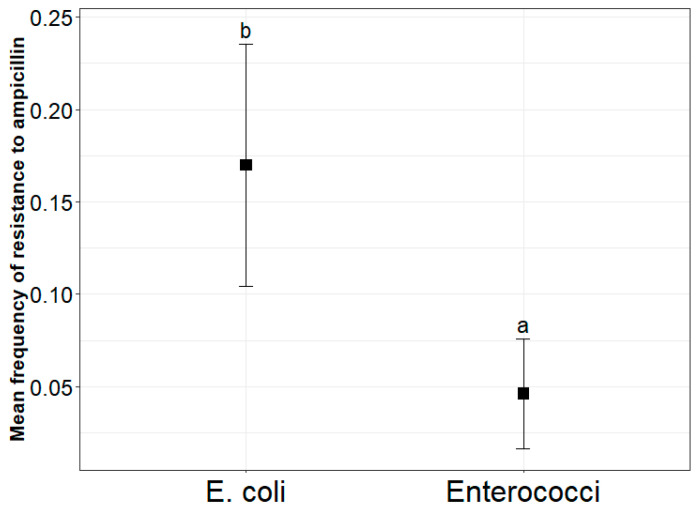
Comparison of the frequency of ampicillin-resistant *E. coli* and enterococci (proportion of total population) with data pooled from all sites. The figure shows estimated least-square (LS) means derived from the fitted beta regression model. Whiskers are the 95% confidence intervals (CIs). Lowercase letters within the plot represent significant differences among groups. The means of groups that do not share a letter are significantly different (*p*-value < 0.05).

*E. coli* was more frequently resistant to ampicillin than enterococci (Figure 1). AmpR *E. coli* across all sites represented 18% of total *E. coli*, while ampR enterococci represented 4% of total *Enterococcus* spp. FIB type (*E. coli* versus enterococci) significantly predicted the frequency of resistance to ampicillin (β = 1.4, *p*-value = 0.0001). Sampling site was not a significant predictor of ampicillin resistance (β = −2.2, *p* = 0.1788) in the beta regression model.

**Figure 2:**
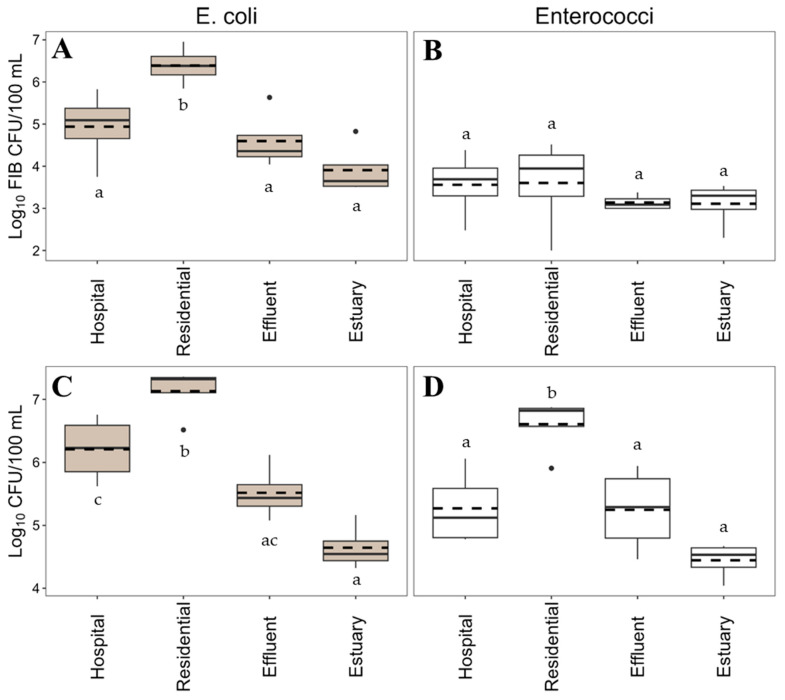
Comparison of log_10_ mean concentrations (log_10_ CFU/100 mL) of ampicillin-resistant *E. coli* (panel (**A**)) and ampicillin-resistant enterococci (panel (**B**)), total *E. coli* (panel (**C**)) and total enterococci (panel (**D**)) among sampling sites. Boxplots represent 1st, median, and 3rd quartiles, and the dotted line represents the mean. Whiskers are the minimum and maximum and dots are outliers. Lowercase letters within the boxplots represent significant differences among sites. The means of sites that do not share a letter within each panel are significantly different (*p*-value <0.05), e.g., a vs. b in panel (**A**).

Mean log_10_ concentrations of ampR *E. coli,* but not ampR enterococci, varied among sites. AmpR *E. coli* concentrations in residential wastewater (log_10_ 6.4 CFU/100 mL) were significantly greater than those at any other site (Figure 2, statistical values in Tables S1 and S2). Total *E. coli* followed the same pattern (Figure 2), as significantly higher concentrations were measured in residential wastewater compared to all other sites. Total *E. coli* concentrations in the hospital wastewater were significantly higher than total *E. coli* concentrations in the estuary as well. No significant differences in mean log_10_ concentrations of ampR enterococci were observed among sites (Figure 2, Table S1). Mean concentration of ampR enterococci among all sites ranged from log_10_ 3.1 to 3.6 CFU/100 mL. Total enterococci concentrations varied among sites, with significantly higher concentrations detected in the residential wastewater compared to all other sites (Figure 2).

### 2.2. Susceptibility of Ampicillin-Resistant in E. coli and Enterococcus spp. to Other Antibiotics

**Figure 3:**
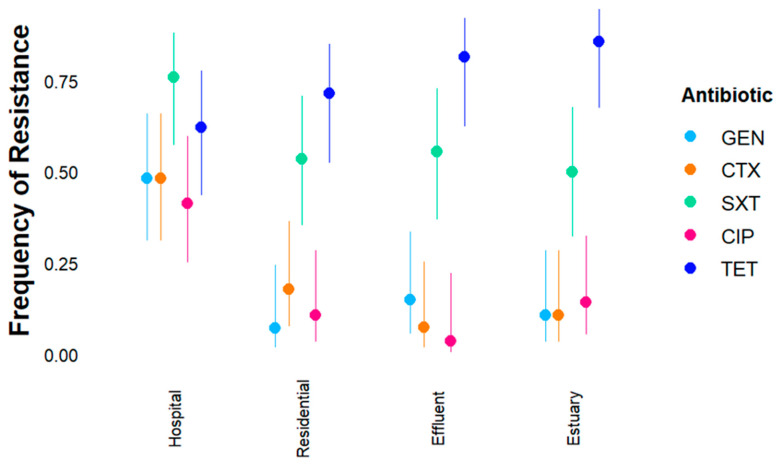
Frequency of additional resistance phenotypes in ampicillin-resistant *E. coli* isolates by site. GEN (gentamicin), CTX (cefotaxime), SXT (trimethoprim-sulfamethoxazole), CIP (ciprofloxacin), and TET (tetracycline). We fitted a logistic model to predict resistance (yes/no) with site, antibiotic, and the interaction effect between site * antibiotic. Frequency of resistance (dots) and confidence intervals (CI) are shown. Amoxicillin + clavulanate was tested but not included in the logistic regression model due to the low frequency of resistance observed.

**Figure 4:**
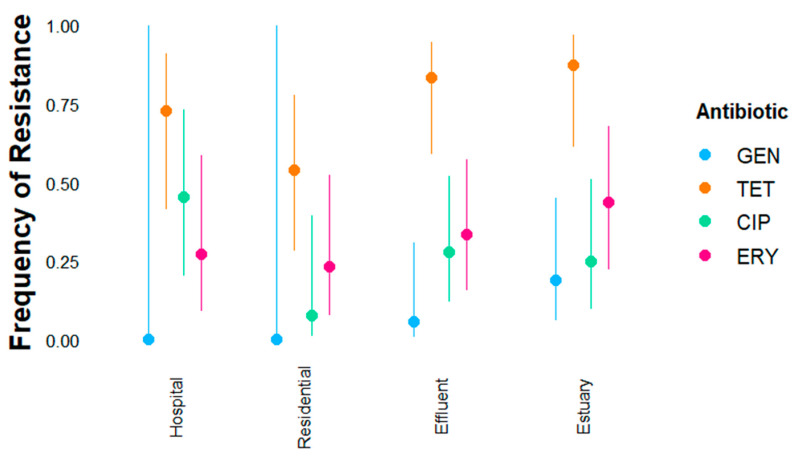
Frequency of additional resistance phenotypes in ampicillin-resistant *Enterococcus* spp. isolates by site for TET (tetracycline), CIP (ciprofloxacin), and ERY (erythromycin). We fitted a logistic model to predict resistance (yes/no) with site, antibiotic, and the interaction effect between both factors (site * antibiotic). Predicted frequency of resistance (dots) and confidence intervals (CIs) are shown. Resistance to gentamicin was not observed in the hospital or residential wastewater, with small sample size resulting in broad confidence intervals (i.e., high uncertainty). Linezolid and vancomycin were tested but were not included in the logistic regression model because resistance was not observed at any site.

Ampicillin-resistant *E. coli* (n = 112) and *Enterococcus* spp. (n = 58) isolates were tested for resistance against six additional antibiotics each (*E. coli*: gentamicin, amoxicillin with clavulanate, cefotaxime, ciprofloxacin, cefotaxime, trimethoprim-sulfamethoxazole, and tetracycline; *Enterococcus*: gentamicin, linezolid, vancomycin, tetracycline, erythromycin, ciprofloxacin). The frequency of *E. coli* and *Enterococcus* spp. isolates that were susceptible, intermediate, or fully resistant to each antibiotic is shown by site (Figures S1 and S2, Tables S3 and S4). All analyses below considered only fully resistant isolates in the “resistant” category unless otherwise noted. Most *E. coli* (75%) and *Enterococcus* spp. (77%) isolates were resistant to tetracycline (Figure 3 and Figure 4), and the majority of *E. coli* isolates were also resistant to trimethoprim-sulfamethoxazole (59%). Only 2% of *E. coli* isolates were resistant to amoxicillin + clavulanate (Figure S1, Table S3), and no *Enterococcus* isolates were resistant to vancomycin or linezolid (Figure S2, Table S4); therefore, these antibiotics were not included in the logistic regression model. Intermediate resistance was observed more often in *Enterococcus* than in *E. coli* (Figures S1 and S2, Tables S3 and S4), and most frequently for ciprofloxacin and erythromycin.

The interaction model (Resistance ~ Site * Antibiotic) without a random effect had the lowest AIC for the *E. coli* isolates (Table S5). The frequency of additional resistance phenotypes observed in ampR *E. coli* isolates was significantly different among antibiotic tests and sites (Figure 3, Table 1), and a significant interaction effect between site and antibiotic was identified (Table 1). The regression model explained 31% of the variance, and the model significantly predicted frequency of resistance (*p*-value = 2.2 × 10^−16^). The interaction between site and antibiotic is clear in the elevated resistance to most antibiotics in hospital isolates compared to isolates from other sites (Figure 3, Tables S6 and S7). *E. coli* isolates from hospital wastewater had a greater likelihood of being resistant to the antibiotics tested compared to other sites (Tables S6 and S7). Frequency of resistance to gentamicin, cefotaxime, and ciprofloxacin was low (<20%) at most sites, except for the hospital wastewater (>40%) (Figure S1, Table S3). The predicted resistance for isolates from the hospital wastewater was significantly elevated for most antibiotics with the exception of tetracycline (Figure 3, Table S6). Despite generally elevated resistance, *E. coli* isolates from the hospital were significantly less likely to be resistant to tetracycline (log odds [Hospital]*[TET] −2.92) compared to isolates from the intercept (Figure 4, Table S6). Resistance to tetracycline was common (>60%) at all sites, as well as resistance to trimethoprim-sulfamethoxazole (>50%), and resistance to these antibiotics was significantly more frequent than resistance to all other antibiotics (Table S7).

**Figure 5:**
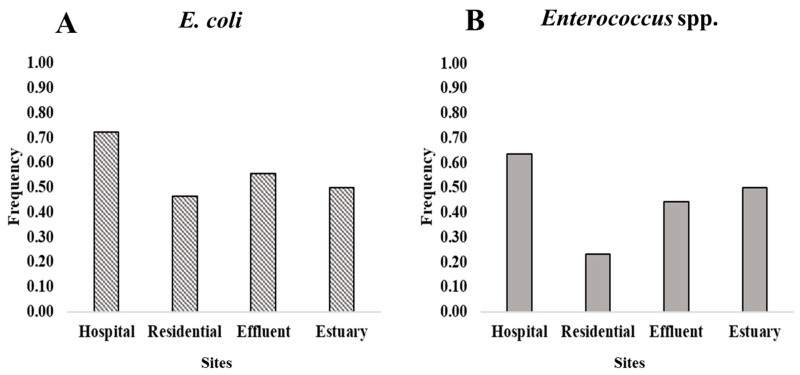
Frequency of multidrug resistance in ampicillin-resistant *E. coli* (n = 112) (panel (**A**)) and *Enterococcus* (n = 58) (panel (**B**)) isolates by site. Sample size per site for *E. coli* is as follows: hospital influent (n = 27), residential influent (n = 29), effluent (n = 28), and estuary (n = 28). Sample size per site for *Enterococcus* is as follows: hospital influent (n = 11), residential influent (n = 13), effluent (n = 18), and estuary (n = 16).

The additive model (Resistance ~ Site + Antibiotic) without the random effect had the lowest AIC for the *Enterococcus* spp. isolates (Table S5). Site and antibiotic were significantly associated with the frequency of resistance in *Enterococcus* isolates, but no significant interaction effect was found between sampling sites and antibiotics (*p*-value = 0.59) (Figure 4, Table 1). The regression model explained 31% of the variance, and the model significantly predicted frequency of resistance (*p*-value = 7.8 × 10^−15^). *Enterococcus* isolates from the estuary (log odds site[Estuary] = 1.50) and the treated effluent (log odds site[Effluent] = 1.13) were significantly more likely to have resistance to additional antibiotics than isolates from the residential wastewater or the hospital (Table S8), e.g., only isolates from the effluent and estuary were gentamicin-resistant (Figure 4 and Figure S2, Table S4). Resistance to tetracycline was common at all sites (overall frequency 74.5%, Figure 5 and Figure S2, Table S4), and resistance to tetracycline was significantly more frequent compared to resistance to all other antibiotics (Tables S8 and S9). There was no difference in predicted frequency between ciprofloxacin and erythromycin, but resistance to both antibiotics was significantly more frequent than resistance to gentamicin (Tables S8 and S9).

### 2.3. Multidrug Resistance in Wastewater, Effluent, and the Estuary

More than 50% of ampR *E. coli* and more than 40% of ampR *Enterococcus* isolates were multidrug resistant (resistant to three or more antibiotic classes) (Figure 5 and Figure S3). The frequency of MDR *E. coli* and *Enterococcus* was highest in hospital wastewater (72% and 63% of ampR isolates, respectively) compared to other sites (Figure 5). *E. coli* and *Enterococcus* isolates from the hospital wastewater were more likely to exhibit multidrug resistance than those from other sites (log odd site [hospital] = 3.63) (Table 2). The regression model for multidrug resistance by sampling site explained only 6% of the variance, indicating that additional factors not considered in the study influenced the frequency of multidrug resistance; however, the model significantly predicted frequency of resistance (*p*-value = 0.037).

*E. coli* isolates from hospital wastewater had the greatest number of resistance phenotypes (to six antibiotics) (n = 7), and this multidrug resistance pattern was the most common pattern for *E. coli* isolated from hospital wastewater (AMP + TET + SXT + GEN+ CIP + CTX) (Table S10). Two multidrug resistance patterns to five antibiotics each were observed in isolates from the hospital (AMP + CTX + AMC + TET + SXT and AMP + GEN + CIP + TET + SXT) (Table S10). The most common multidrug resistance pattern observed in *E. coli* isolates (n = 26, 40.6% of all MDR *E. coli*) was resistance to ampicillin, tetracycline, and trimethoprim-sulfamethoxazole (Table S10). Two multidrug resistance patterns were equally prevalent in *Enterococcus*: resistance to ampicillin, ciprofloxacin, and tetracycline (n = 10, 41.6% of all MDR *Enterococcus*), and resistance to ampicillin, tetracycline, and erythromycin (n = 10, 41.6% of all MDR *Enterococcus*) (Table S11).

The *Enterococcus* isolate with the greatest number of resistance phenotypes among all *Enterococcus* (to five antibiotics) (n = 1) was isolated from the estuary (Figure S3, Table S11). This isolate was resistant to AMP, GEN, CIP, TET, and ERY. Three *E. coli* colonies that were also resistant to five antibiotics were isolated from the estuary (Figure S3). These three isolates all shared a multidrug resistance pattern (AMP + GEN + CIP + TET + SXT) (Table S10), which was also observed in isolates from the hospital.

## 3. Discussion

Antibiotic resistance is a global threat, yet low- and middle-income countries like Costa Rica may shoulder a disproportionate burden. These areas face unique socioeconomical challenges, the frequency of antibiotic resistance is typically higher, and the efficacy of wastewater treatment varies greatly [43,44,45]. The WWTP sampled in this study carried out secondary (biological) treatment but not disinfection and, as a result, discharged a high load of fecal microorganisms and ARB into the estuary daily. The frequency of antibiotic and multidrug resistance in tropical environments is poorly explored [27]; this study supplements the current information, and demonstrates a significant difference between the frequency of ampR *E. coli* and enterococci, a high frequency of resistance for both ampR FIB types to tetracycline and other antibiotics, as well as higher incidence of multidrug resistance in samples from the hospital wastewater compared to other sites. The results of this study also point to the estuary as an area of concern, as *E. coli* and *Enterococcus* isolates with resistance to at least five antimicrobial classes were found in the estuary.

### 3.1. Resistance of E. coli and Enterococci to Ampicillin in Wastewater, Effluent, and the Puntarenas Estuary

Ampicillin resistance frequencies of FIB isolated from wastewater and surface waters in this study (∼18% for *E. coli* and ∼4% for *Enterococcus*) were within the range of those reported in studies from other regions. For example, resistance to ampicillin in *E. coli* was 19% in Japan [46] and 5% in the United States [47]. The frequency of resistance to ampicillin in *Enterococcus* in European countries (Greece, Poland, and Netherlands) was reported at between 16% and 7% [48,49,50,51]. A study on ampicillin-resistant FIB in San José, Costa Rica found that 57% of *E. coli* isolated from freshwater upstream and downstream of a hospital discharge were resistant to ampicillin, but intermediate and full resistance were combined into one “resistant” category [52]. Similarly, a study conducted in Poland found resistance to ampicillin in 34% of *E. coli* isolates but also combined intermediate and full resistance [48].

We hypothesized that the frequency of ampicillin resistant *E. coli* and *Enterococcus* would be highest in the hospital wastewater compared to residential wastewater and environmental samples, but no significant difference by site was found. Antibiotic use in hospitals is typically elevated compared to residential areas, so it would be expected to see that reflected in the wastewater. Similarly, a Romanian study found no significant difference in the frequency of ampR *E. coli* in hospital vs. community wastewater [53]. A study in France found significantly greater proportions of ampR *Enterococcus* in wastewater from a hospital (100%) compared to other sources, which included wastewater from a retirement home (87.5%), combined hospital and community wastewater (19.1%), treated effluent (19.0%), and the river where the effluent is discharged (4.0%) [54]; however, only one sample per site was analyzed.

The frequency of ampR *E. coli* was significantly higher than the frequency of ampR *Enterococcus.* This is comparable to other studies. A study in Poland found a higher percentage of *E. coli* were resistant to ampicillin compared to *Enterococcus* (34% compared to 7%) [48]. A study performed in the USA that grouped intermediate and fully resistant isolates into the “resistant” category found ampicillin resistance in *E. coli* isolates was between 17.2% and 48.7%, depending on the site, while ampicillin resistance in *Enterococcus* isolates was between 0% and 2.3% [55].

The comparisons made above come with a caveat: antibiotic resistance studies, particularly those carried out in the environment, do not employ consistent methodological or reporting standards, hindering valid comparisons among studies. Our data on antibiotic resistance reports only full resistance, but some studies, such as Łuczkiewicz et al., 2010, Garcia et al., 2007, and Tzoc et al., 2004, combine intermediate and full resistance into one “resistant” category, resulting in reporting of relatively high frequency of antibiotic-resistant isolates. Many studies do not employ a standard method for isolating FIB, e.g., the XM-G agar employed in Ma et al., 2022. Antibiotic-resistant isolates should be confirmed to genus or species, and this is particularly important when isolation methods that are not standardized, or standard methods with a known high error rate such as ISO 7899-2(2000) [56] are employed. A related issue is that some antibiotic resistance data are based on the European Committee on Antimicrobial Susceptibility (EUCAST) criteria, while others are based on CLSI Guidelines, which specify quite different breakpoints in some cases.

The concentration of ampR *E. coli* observed in this study was significantly higher in residential wastewater compared to other sites, while no difference in mean concentrations of ampR by site for *Enterococcus* spp. was detected. The high concentration of ampR *E. coli* in the residential wastewater was influenced by the higher total *E. coli* concentration at that site, as the frequency of ampicillin resistant *E. coli* did not differ significantly by site. Studies that reported concentrations of ampR *E. coli* in Ireland [57] and ampR *Enterococcus* in Chile [58] found no significant differences between hospital and residential sewage.

The high concentration of ampR FIB in the treated wastewater effluent in this study (4.59 log10 CFU/100 mL *E. coli*, 3.14 log10 CFU/100 mL *Enterococcus*) can be explained by the lack of a disinfection step prior to releasing the secondary-treated effluent into the environment at El Roble WWTP. Higher-income countries like the United States, Japan, and some European countries typically include a disinfection step to eliminate the bacterial load before releasing the treated effluent into the environment [49,59,60], which generally results in at least a 2-log reduction of FIB after disinfection of treated effluent with chlorine [48,49]. The USA uses NPDES permits to set the acceptable level of bacterial discharge from WWTPs [61]. *E. coli* levels are generally undetectable, or less than 10 CFU/100 mL [62,63].

### 3.2. Susceptibility of Ampicillin-Resistant E. coli and Enterococcus to Other Antibiotics

Only 7.1% of ampR *E. coli* and 12.1% of ampR *Enterococcus* did not exhibit resistance to any other antibiotics tested in this study. As expected, a high proportion of ampR FIB were resistant to the older antibiotics, such as tetracycline and trimethoprim-sulfamethoxazole. Estimates of national consumption of antibiotics in Costa Rica, which were calculated from importation manufacture data, showed ciprofloxacin, trimethoprim-sulfamethoxazole, cefotaxime, and macrolides are frequently consumed, with several hundred doses per 1000 inhabitants consumed daily [8,64]. Tetracyclines are commonly used in the food industry (i.e., pigs, chicken, and tilapia) and gentamicin was one of the main imports used for crop production in Costa Rica [8,65]. The high frequency of ciprofloxacin-resistant *E. coli* and *Enterococcus,* and erythromycin-resistant *Enterococcus* observed in this study was particularly concerning, as these antibiotics are first-line drugs for many clinical applications [1,41].

Resistance to several antibiotics used in this study was relatively infrequent. No resistance to linezolid and vancomycin was detected in ampR *Enterococcus.* AmpR *E. coli* was rarely resistant to amoxicillin + clavulanate. Usage of linezolid and vancomycin was not quantified in the estimates of national consumption, which may indicate limited use; however, official reporting may not capture all antibiotic usage. Usage of amoxicillin + clavulanate was frequent, with a higher average daily dose per 1000 inhabitants (7.25) than all third-generation cephalosporins and ciprofloxacin combined (4.53) in 2022 [64], yet resistance was rare. Amoxicillin + clavulanate belongs to both the penicillin and beta-lactamase antibiotic groups, making it more difficult for bacteria to develop resistance against it.

No *Enterococcus* isolated in this study were resistant to vancomycin, yet previous studies in Costa Rica suggest that vancomycin-resistant *Enterococcus* is of concern. One study conducted in Costa Rica found VRE in 52% of patients at two hospitals in San Juan; however, samples were only taken from patients in intensive care units, and intermediate and full resistance were combined [66]. A survey of Costa Rican livestock found VRE in the feces of 13% of animals sampled [67]. The glycopeptide avoparcin, which is known to select for VRE [68], was used as a growth promoter for livestock in Costa Rica until 2000 [67]. While a low frequency of resistance of *Enterococcus* spp. to vancomycin has been reported in several Central and South American countries in the past [69,70], VRE are of concern in many regions [71,72,73,74].

Resistance to trimethoprim-sulfamethoxazole has been widely documented in Central America and Costa Rica [75,76,77,78]. It is a relatively inexpensive drug that has been in use for many decades and is widely available in low- and middle-income countries to treat various infections [79]. In this study, almost 60% of *E. coli* isolates exhibited resistance to trimethoprim-sulfamethoxazole. This falls within the estimation by the International WhoNET surveillance program that, out of >20,000 *E. coli* isolates, 41 to 62% of isolates from central America and Asia were resistant to trimethoprim-sulfamethoxazole, versus 9 to 23% of isolates that were resistant in the US and Sweden [79].

Resistance of *E. coli* to cefotaxime, a third-generation cephalosporin, was more frequent in hospital wastewater than in residential wastewater, treated effluent, and the estuary. *E. coli* resistance to third generation cephalosporins has been increasing in hospital and community settings in Europe [80]. The European Antimicrobial Resistance Surveillance Network (EARS-Net) reported an increase in resistance (∼15%) of *E. coli* to third-generation cephalosporins in bacteremia in the hospital setting during 2017 [80]. Resistance to third-generation cephalosporins is considered a high community and health-care burden by the World Health Organization [81]. The worldwide estimate in 2020 of the percentage of bloodstream infections due to *E. coli* resistant to third-generation cephalosporins was 42% [82].

### 3.3. Multidrug Resistance of E. coli and Enterococcus in Wastewater, Effluent, and the Estuary

More than 50% of all ampR *E. coli* isolates and 40% of all ampR *Enterococcus* isolated in this study exhibited multidrug resistance. Other studies of antibiotic resistance in wastewater found comparable frequencies, albeit from total FIB concentrations instead of ampR FIB concentrations; a study in Romania found that 80.2% of total *E. coli* isolates exhibited multidrug resistance, and that there was no difference in prevalence between hospital and community wastewater sources of isolates [53]. Studies in Canada and South Africa found 23.6% [83] and 68% [84], respectively, of total *Enterococcus* isolates exhibited multidrug resistance.

Although residential wastewater had the highest concentration of ampR *E. coli,* the likelihood of multidrug resistance in *E. coli* and *Enterococcus* isolates was higher in hospital wastewater compared to residential wastewater, treated effluent, and the estuary. AmpR *E. coli* and *Enterococcus* isolates from hospital sewage exhibited high frequencies of multidrug resistance (70% and 63%, respectively). The frequency of multidrug resistance in total *E. coli* isolates from hospital sewage was 29.7% in the USA [85], 32% in Vietnam [86], and 85% in Romania [53]. The frequency of multidrug resistance in total *Enterococcus* isolates from hospital sewage was 57.6% in Iran [87] and 94% in Poland [88]. The variation of multidrug resistance frequencies may be due to regional differences, such as infrastructure and antibiotic use.

**Figure 6:**
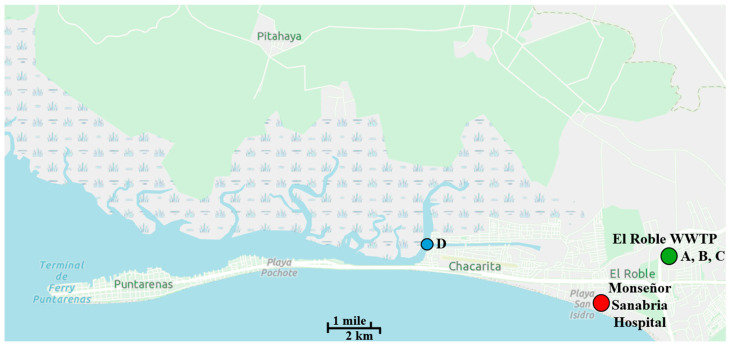
Map of the study site. El Roble Wastewater Treatment Plant (WWTP) 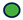 and Monseñor Sanabria Hospital 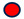 appear on map. Sampling stations are designated: (A) hospital wastewater, (B) residential wastewater, (C) treated effluent, and (D) Puntarenas estuary 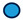 where the WWTP effluent is discharged. A scale is provided at the bottom of the map.

This study found that *Enterococcus* spp. isolates from the estuary and the treated effluent were more likely to be resistant to additional antibiotics than isolates from untreated hospital and residential wastewater. Three *E. coli* isolates and one *Enterococcus* isolate from the estuary were resistant to at least five antibiotics. In the case of *Enterococcus,* the isolate from the estuary displayed more resistance phenotypes than any other. The discharge of the El Roble WWTP is probably not the only contributor to the high frequency of antibiotic resistance in the estuary. Puntarenas Estuary also receives untreated wastewater from household onsite wastewater treatment systems [89]. An agricultural region to the north (Figure 6) where cattle are grazed may also contribute ARB to the estuary via runoff.

## 4. Materials and Methods

### 4.1. Study Site

Wastewater samples were collected in the city of Puntarenas on the central Pacific coast of Costa Rica (Figure 6). The population of the city of Puntarenas was 141,000 in 2022 [90]. Wastewater from several residential areas and Monseñor Sanabria Hospital is treated in El Roble WWTP. After secondary treatment, and without disinfection, the effluent is discharged into Puntarenas Estuary, which drains into the Gulf of Nicoya (Figure 6). Several upgrades have been made to El Roble WWTP since it was built. Last published data indicated that the plant has a flow of 5,011,200 L per day and uses activated sludge technology [91]. Four sampling sites were chosen: the untreated wastewater from the hospital, the wastewater from one of the residential areas, the treated effluent discharged from the WWTP (9°58′54.3″N, 84°44′18.8″W), and Puntarenas Estuary (9°59′02.0″N, 84°46′54.9″W), which receives the effluent.

### 4.2. Sampling and Culture of FIB

Samples of 500 mL were collected at each of the four sites in Puntarenas, Costa Rica on four occasions between October and December 2019. At El Roble WWTP, samples were collected from the influent from the hospital and influent from the residential areas, and also from the treated effluent (Figure 6) using sterile 500 mL containers. The hospital and residential influent samples were collected from dedicated pipes from the Monseñor Sanabria Hospital and local residential area, respectively. The treated effluent samples were collected from the clarified water outlet, post-secondary treatment. The estuary was sampled from the shore with an open sampling container attached to a rope that was thrown 5 m into the estuary. The container was pulled back to shore, and the water was immediately poured into a sterile container. The samples were transported on ice to the Laboratorio Nacional de Aguas (National Water Laboratory) in Tres Rios, Cartago, Costa Rica. There, the bacteria were concentrated by membrane filtration following US EPA Method 1603 for enumeration of *E. coli* [92] and US EPA Method 1600 for enumeration of enterococci [42]. The volume that was filtered (1, 10, or 100 mL) varied depending on the sampling site, and 1:10 dilutions were made, when necessary, to obtain countable numbers of colonies. After membrane filtration, filters were placed on mTEC agar plates without antibiotic to measure total *E. coli,* and on mTEC amended with 16 µg/mL ampicillin to measure *E. coli* with intermediate resistance to ampicillin based on Clinical and Laboratory Standards Institute (CLSI) guidelines [41]. Filters were placed on mEI agar plates without antibiotic to measure total enterococci, and on mEI amended with 16 µg/mL ampicillin to measure ampR enterococci. Ten colonies per site for each FIB were selected from ampicillin-amended plates at each sampling event and subcultured to brain heart infusion (BHI) with ampicillin (16 µg/mL), and ampR FIB were stored individually in cryovials containing 50% glycerol. These cryovials were stored at 4 °C for a period of ≈ 2 weeks and shipped at room temperature to the University of South Florida (USF) for further antibiotic resistance testing.

### 4.3. Phylogenetic Confirmation of Ampicillin-Resistant Isolates

AmpR isolates were stored at −80 °C upon receipt at USF. Before further testing, the putative ampR *E. coli* and *Enterococcus* spp. were subcultured three times on brain heart infusion agar with ampicillin to ensure isolation of a pure culture. An isolated colony was picked from the most recent subculture and resuspended in 50 µL of nuclease free water, which was then boiled to extract the DNA via the boil lysis method. Polymerase chain reaction (PCR) of the *uidA* gene, which is specific to *E. coli*, was used to confirm the species of putative *E. coli* isolates [92]. qPCR for the 23S rRNA gene was used to confirm the genus of putative *Enterococcus* spp. isolates [93]. Due to shipping and a freezer malfunction, fewer than the anticipated 160 putative *E. coli* and *Enterococcus* isolates were recovered. All putative ampR *E. coli* (n = 116) and *Enterococcus* spp. (n = 58) isolates were confirmed to species or genus, respectively. Confirmed isolate number per site for *E. coli* and *Enterococcus* was as follows: hospital influent (n = 27 *E. coli* and 11 *Enterococcus*), residential influent (n = 29 and 13), effluent (n = 28 and 18), and estuary (n = 28 and 16).

### 4.4. Multidrug Resistance Testing

The susceptibility of isolates to additional antibiotic classes was determined using the Kirby–Bauer disc diffusion assay, and resistance to ampicillin was also confirmed by this procedure. The antibiotics used for the Kirby–Bauer disc diffusion assay were chosen from the CLSI 2020 [41] (Tables S12 and S13). Multiple antibiotic classes were selected, which included antibiotics that have clinical relevance or that are frequently used in Costa Rica. *E. coli* isolates were tested against ampicillin, ciprofloxacin, cefotaxime, amoxicillin with clavulanate, gentamicin, tetracycline, and trimethoprim-sulfamethoxazole, while *Enterococcus* spp. isolates were tested against ampicillin, ciprofloxacin, vancomycin, tetracycline, linezolid, gentamicin, and erythromycin.

Isolates that had been stored in the −80 °C freezer after confirmation as *E. coli* or *Enterococcus* spp. were recovered onto BHI plates amended with 16 µg/mL ampicillin [41]. Isolated colonies were picked using an inoculating loop and placed into 1 mL of a 0.85% saline solution. The density of bacteria in the solution was measured using a Nanodrop instrument, by measuring absorbance at 625 nm [94]. If necessary, saline or bacteria were added to achieve the ideal absorbance range (0.08–0.13 AU). Immediately following the absorbance measurement, a sterile swab was dipped into the prepared inoculum and streaked over a prepared Mueller-Hinton plate [94]. Antibiotic discs were placed on the plates using sterile forceps and incubated at 35 °C for 24 h. The size (in millimeters) of each zone of inhibition was measured and recorded. The resistance or susceptibility of the bacteria to each antibiotic was determined according to CLSI standards [41]. Although *E. coli* and *Enterococcus* were isolated using ampicillin concentrations representing intermediate and full resistance, respectively (see methods section *Sampling and culture of FIB*), all *E. coli* isolates tested for multidrug resistance (n = 112) were confirmed to be fully ampicillin resistant, hence will be referred to as ampicillin-resistant *E. coli* isolates in this study.

### 4.5. Statistical Analysis

#### 4.5.1. Frequency of Resistance of *E. coli* and *Enterococci* to Ampicillin

We calculated the frequency (proportion) of ampR *E. coli* and enterococci at each site as the ratio between the concentration of bacteria on ampicillin-amended plates (ampicillin-resistant) divided by the concentration on unamended plates (total). We explored the relationship between the frequency of resistance to ampicillin (dependent variable) and FIB group (*E. coli* or *Enterococcus*) and sampling sites (independent variables) by means of a beta regression analysis using the betareg package [95]. A new variable, frequency, was calculated as the ratio between the concentration of *E. coli* or enterococci on ampicillin-amended plates (ampicillin-resistant) divided by the concentration on unamended plates (total) for each observation. Since the frequency of resistance of FIB to ampicillin ranges between 0 to 1, the model assumes that the data follows a beta distribution. Pseudo-R-squared produced by the summary function in betareg were used as a measure for goodness of fit, and the test for the *p*-value for the model was produced using the lrtest function in the lmtest package [96]. Lastly, after fitting the model we performed a post hoc comparison among groups by estimating and comparing marginal means (Least-Squares Means) derived from the model.

#### 4.5.2. Concentrations of Ampicillin-Resistant *E. coli* and *Enterococci*

Concentrations of ampR *E. coli* and enterococci were compared using a one-way ANOVA to determine if there were significant differences in ampR FIB concentrations among sampling sites. FIB data were log_10_-transformed to approximately conform to a normal distribution. Groups being compared were determined to have equal variance using the Bartlett test before performing ANOVA analysis. Tukey’s HSD test, which accounts for the probability of making one or more Type I errors, was used as post hoc analysis to test for significant differences in all pairwise site comparisons.

#### 4.5.3. Susceptibility of Ampicillin-Resistant FIB to Other Antibiotics

Ampicillin-resistant *E. coli* isolates were tested for susceptibility to ciprofloxacin, cefotaxime, amoxicillin with clavulanate, gentamicin, tetracycline, and trimethoprim-sulfamethoxazole, while ampR *Enterococcus* spp. isolates were tested against ciprofloxacin, vancomycin, tetracycline, linezolid, gentamicin, and erythromycin. A binary variable was created where fully resistant isolates were designated with a 1, and intermediate resistant and susceptible isolates were designated with a 0. Residential wastewater and gentamicin were chosen as the site and antibiotic intercepts, due to lower resistance for both compared to other sites and antibiotics, and because gentamicin was tested against both FIB types.

We conducted model selection among various formulations of logistic regression models to assess the relationship between FIB antibiotic resistance frequency, sampling site, and antibiotic. Analysis was conducted separately on *E. coli* and *Enterococcus* data. For each dataset we fit an additive model (sampling site + antibiotic), an interaction model (sampling site + antibiotic + sampling site * antibiotic) and the interaction model with a random effect of sampling day to account for the non-independence of samples taken on the same day. The Akaike information criterion (AIC) was used to compare different possible models and assess the necessity of including the random effect. Tjur’s coefficient of discrimination was used as a measure for goodness of fit [97]. Log-odds for the interaction terms and the 95% CIs were calculated for *E. coli* using base R functions coef and confint. Amoxicillin + clavulanate was not included in the logistic regression model for *E. coli*, and linezolid and vancomycin were not included for *Enterococcus* spp. due to the high level of susceptibility to these antibiotics across all sites (85–100% of isolates per antibiotic). A Tukey HSD test was used as post hoc analysis for significant differences in the likelihood for additional resistance for each of the variables (site and antibiotic) in pairwise comparisons.

Multidrug resistance was defined as resistance of a given isolate to 3 or more antibiotic classes. We compared the frequency of multidrug resistance between FIB and among sites by first creating a new binary variable, where MDR FIB isolates were designated with a 1 (resistant to three or more antibiotic classes) and isolates resistant to less than 3 antibiotic classes were designated with a 0. We fitted a linear logistic regression model (estimated using maximum likelihood) to predict frequency of multidrug resistance with sampling site (formula: multidrug resistance ~ site + FIB type). An analysis of deviance was performed to compare how much the regression model improved by adding the predictor when compared to the null model (a model without predictors) and calculated a *p*-value to test if the independent variables provide a statistically significant improvement on the null model. The Cragg–Uhler (Nagelkerke) coefficient of discrimination was used as a measure for goodness of fit. Ninety-five percent confidence intervals (CIs) and *p*-values were computed using a Wald z-distribution approximation.

## 5. Conclusions

The data resulting from our study support the hypothesis that wastewater effluent derived from hospital and residential waste is an important source of ARB, including MDR bacteria, in Puntarenas Estuary. These findings highlight the importance of treating and disinfecting wastewater effluent prior to its release into the environment. Costa Rica has already implemented some antibiotic hospital stewardship initiatives [98], and in 2019 the government of Costa Rica passed an executive decree to confront the issue of antimicrobial resistance [99]. The plan focuses on efforts to optimize the use of antibiotics and presents strategies to improve antibiotic stewardship in human, animal, and plant health. However, the action plan does not address ARB in wastewater effluent or surface waters as potential contributing factors to the rise in antibiotic resistance, nor does it include the environmental fate of antibiotics. Further efforts to identify regionally specific patterns of resistance in wastewater and the environment in Costa Rica are likely to improve our understanding of antibiotic resistance. This will help policy makers to develop locally relevant interventions and improve antimicrobial stewardship plans.

## Figures and Tables

**Table 1:** Relationship of frequency of resistance to site and antibiotic in MDR testing. (A) Logistic regression models used separately to predict *E. coli* and *Enterococcus* frequency of resistance with sampling sites, antibiotics tested, and their interaction (sites * antibiotic) and (B) a linear regression model used to predict FIB (*E. coli* and *Enterococcus*) frequency of resistance to three or more antibiotic classes with sampling site and FIB type. Significant relationships are bolded (*p*-values < 0.05).

(A)			
**Model**	**FIB**	**Chisq (χ^2^)**	***p*** **-Value**
Site	*E. coli*	29.076	**<0.001**
	*Enterococcus*	9.862	**0.020**
Antibiotic	*E. coli*	141.95	**<** **0.001**
	*Enterococcus*	71.301	**<0.001**
Site: Antibiotic	*E. coli*	30.312	**0.003**
	*Enterococcus*	7.495	0.586
(B)			
**Model**	**Chisq (χ^2^)**	***p*-Value**	
Site	7.8561	**0.04928**	
FIB	2.0838	0.14887	

**Table 2:** Influence of site and FIB on the log odds of multidrug resistance frequency. The model’s intercept corresponds to site [residential wastewater] or FIB [*E. coli*]. Significant relationships are bolded (*p*-values < 0.05).

Multidrug Resistance			
Predictors	Log Odds	CI	*p*
(Intercept)	0.74	0.38–1.42	0.373
Site [Hospital]	3.63	1.46–9.43	**0.006**
Site [Effluent]	1.71	0.72–4.12	0.224
Site [Estuary]	1.76	0.74–4.26	0.201
FIB [Enterococcus]	0.62	0.32–1.19	0.150
Observations	170		
R^2^ Nagelkerke	0.078		

## Data Availability

Data is available at https://doi.org/10.17632/z7rw9y96rp.1.

## Supplementary Materials

The following supporting information can be downloaded at https://www.mdpi.com/article/10.3390/antibiotics14101024/s1. Figure S1: Frequency of resistance of ampicillin-resistant *E. coli* isolates at each site to the following antibiotics: cefotaxime (CTX), ciprofloxacin (CIP), amoxicillin + clavulanate (AMC), gentamicin (GEN), tetracycline (TET), and trimethoprim-sulfamethoxazole (SXT). Ampicillin is not shown in the plot as all isolates tested were fully resistant to ampicillin, the selective antibiotic in the isolation procedure. The size and color of the circle denotes frequency (larger circle and red color denotes higher frequency). Figure S2: Frequency of resistance of ampicillin-resistant *Enterococcus* isolates to the following antibiotics: ciprofloxacin (CIP), erythromycin (ERY), gentamycin (GEN), linezolid (LZD), tetracycline (TET), and vancomycin (VAN). Ampicillin is not shown in the plot as all isolates tested were fully resistant to ampicillin, the selective antibiotic in the isolation procedure. The size and color of the circle denotes frequency (larger circle and red color denotes higher frequency). Figure S3: Proportion of isolates resistant to multiple classes of antibiotics among *E. coli* (panel A) and *Enterococcus* (panel B) isolates at each site. Sample size per site for *E. coli* and *Enterococcus* is as follows: Hospital influent (n = 27 and 11), residential influent (n = 29 and 13), effluent (n = 28 and 18) and estuary (n = 28 and 16). Color scale indicates number of resistance phenotypes: darker colors indicate more resistance phenotypes. The height of each block within a column indicates the proportion of isolates with a given number of resistance phenotypes. Table S1: Statistical results for testing for differences in log_10_ mean total and ampicillin-resistant *E. coli* and enterococci concentrations among sampling sites. Significant differences (*p*-values < 0.05) are bolded. *p*-values associated with Tuckey HSD test are shown in the last column. Table S2: Significance of pairwise comparisons of log_10_ mean concentrations of ampR *E. coli* by site. Significant differences between sites (*p*-values < 0.05) are bolded. No significant difference among log_10_ mean concentrations of ampicillin-resistant enterococci by site were observed, therefore a post-hoc test was not necessary. Table S3: Frequency of susceptibility of ampicillin-resistant *E. coli* isolates at each site to the following antibiotics: cefotaxime (CTX), ciprofloxacin (CIP), amoxicillin + clavulanate (AMC), gentamicin (GEN), tetracycline (TET), and trimethoprim sulfamethoxazole (SXT). Ampicillin is not shown as all isolates tested were fully resistant to ampicillin, the selective antibiotic in the isolation procedure. Table S4: Frequency of susceptibility of ampicillin-resistant *Enterococcus* isolates to the following antibiotics: ciprofloxacin (CIP), erythromycin (ERY), gentamycin (GEN), linezolid (LZD), tetracycline (TET), and vancomycin (VAN). Ampicillin is not shown as all isolates tested were fully resistant to ampicillin, the selective antibiotic in the isolation procedure. Table S5: Akaike’s information criterion (AIC) values for logistic regression models applied to *E. coli* and *Enterococcus* datasets for susceptibility to additional antibiotics. AIC for the preferred model is in bold. Table S6: Influence of site and antibiotics on the log odds of *E. coli* resistance frequency to additional antibiotics. The model’s intercept corresponds to site [residential wastewater], antibiotic [GEN] or their interaction [residential wastewater] * [GEN]. Significant relationships are bolded (*p*-values < 0.05). The significant *p*-value for the intercept indicates that the predicted resistance is significantly different than 0. Table S7: Post-hoc comparisons of the logistic model on influence of site and antibiotics on the log odds of *E. coli* resistance frequency to additional antibiotics by (A) site and (B) antibiotic. Significant relationships are bolded (*p*-values < 0.05), marginally significant relationships (*p*-values = 0.05) are italicized. Table S8: Influence of site and antibiotic on the log odds of *Enterococcus* resistance frequency to additional antibiotics. The model’s intercept corresponds to site [residential wastewater] and antibiotic [GEN]. Significant relationships are bolded (*p*-values < 0.05). The significant *p*-value for the intercept indicates that the predicted resistance is significantly different than 0. Table S9: Post-hoc comparisons of the logistic model on influence of site and antibiotics on the log odds of *Enterococcus* resistance frequency to additional antibiotics by (A) site and (B) antibiotic. Significant relationships are bolded (*p*-values < 0.05). Table S10: Antibiotic resistant profiles observed in *E. coli* isolates. Sample size (isolate number) per site is as follows: Hospital influent (n = 27), residential influent (n = 29), effluent (n = 28) and estuary (n = 28). Table S11: Antibiotic resistant profiles observed in *Enterococcus* spp. isolates. Sample size (isolate number) is as follows: Hospital influent (n = 11), residential influent (n = 13), effluent (n = 18) and estuary (n = 16). Table S12: Antibiotics chosen for the Kirby-Bauer disc diffusion assay for *E. coli*. Table S13. Antibiotics chosen for the Kirby-Bauer disc diffusion assay for *Enterococcus* spp.

## Abbreviations

AMR	Antimicrobial resistance
ARB	Antimicrobial resistant bacteria
ARG	Antimicrobial resistance genes
CFU	Colony-forming units
FIB	Fecal indicator bacteria
MDR	Multidrug resistant
VRE	Vancomycin-resistant Enterococcus
WHO	World Health Organization
WWTP	Wastewater treatment plant
