# Supplementary material for: Ampicillin- and Multidrug-Resistant Escherichia coli and Enterococcus spp. in Costa Rican Wastewater and Surface Water
Source: Antibiotics (Basel). 2025 Oct 14;14(10):1024. doi: 10.3390/antibiotics14101024 (PMC12561136; doi:10.3390/antibiotics14101024)
Supplement: Supplementary file 1 [file antibiotics-14-01024-s001.zip › antibiotics-3898015-supplementary.pdf]

**Supplementary Materials**

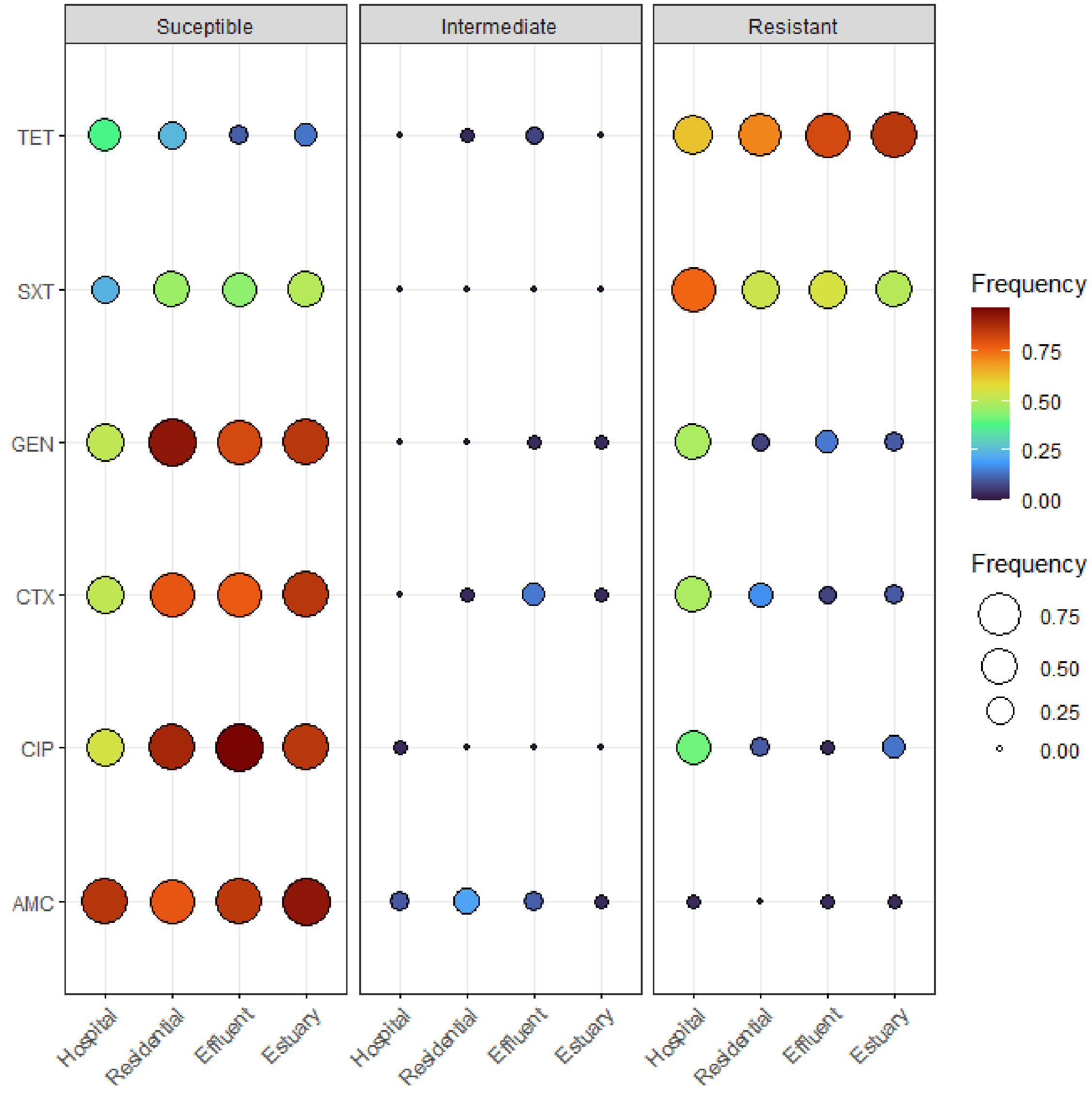


**Figure S1.** Frequency of resistance of ampicillin-resistant *E. coli* isolates at each site to the following antibiotics: cefotaxime (CTX), ciprofloxacin (CIP), amoxicillin + clavulanate (AMC), gentamicin (GEN), tetracycline (TET), and trimethoprim-sulfamethoxazole (SXT). Ampicillin is not shown in the plot as all isolates tested were fully resistant to ampicillin, the selective antibiotic in the isolation procedure. The size and color of the circle denotes frequency (larger circle and red color denotes higher frequency).

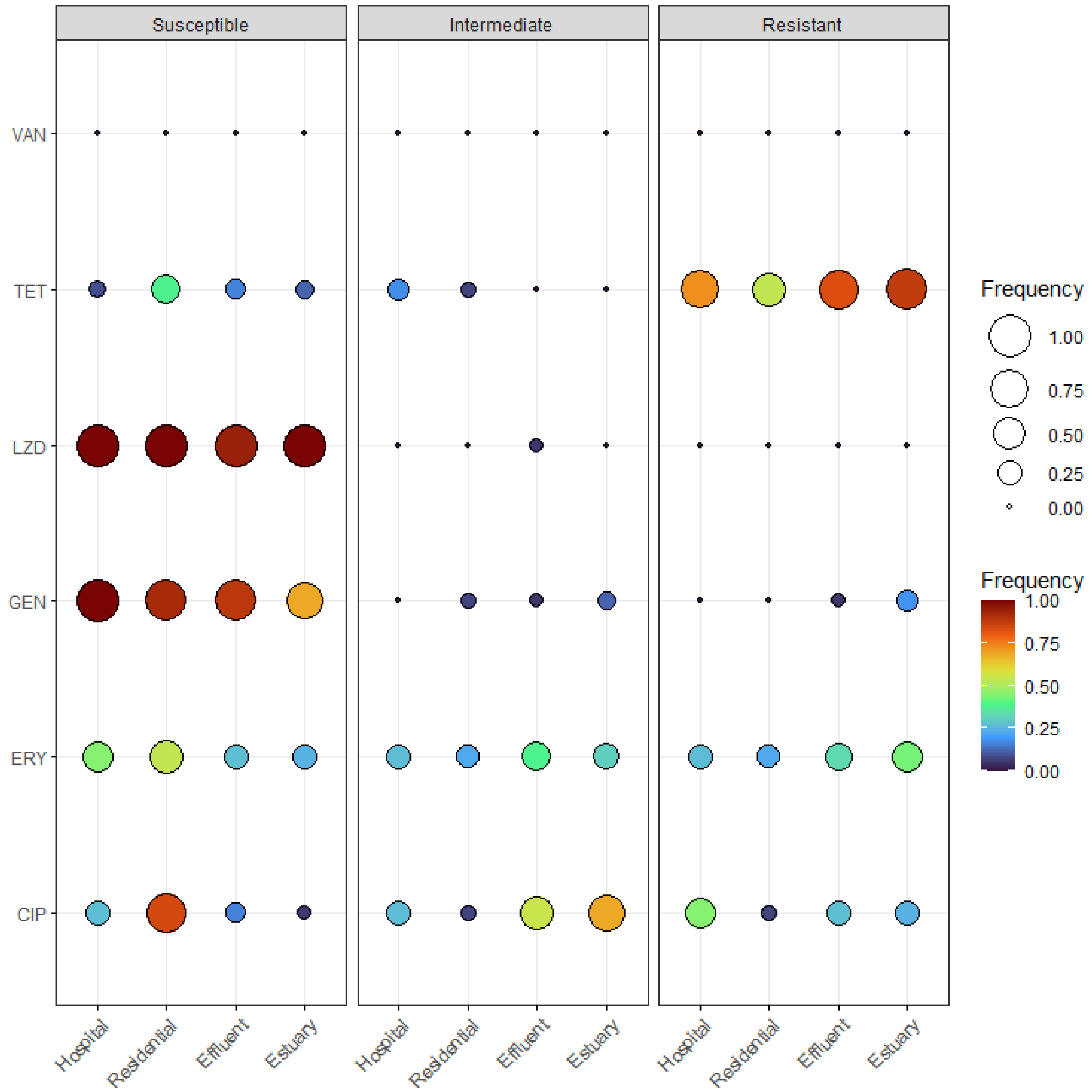


**Figure S2.** Frequency of resistance of ampicillin-resistant *Enterococcus* isolates to the following antibiotics: ciprofloxacin (CIP), erythromycin (ERY), gentamycin (GEN), linezolid (LZD), tetracycline (TET), and vancomycin (VAN). Ampicillin is not shown in the plot as all isolates tested were fully resistant to ampicillin, the selective antibiotic in the isolation procedure. The size and color of the circle denotes frequency (larger circle and red color denotes higher frequency).

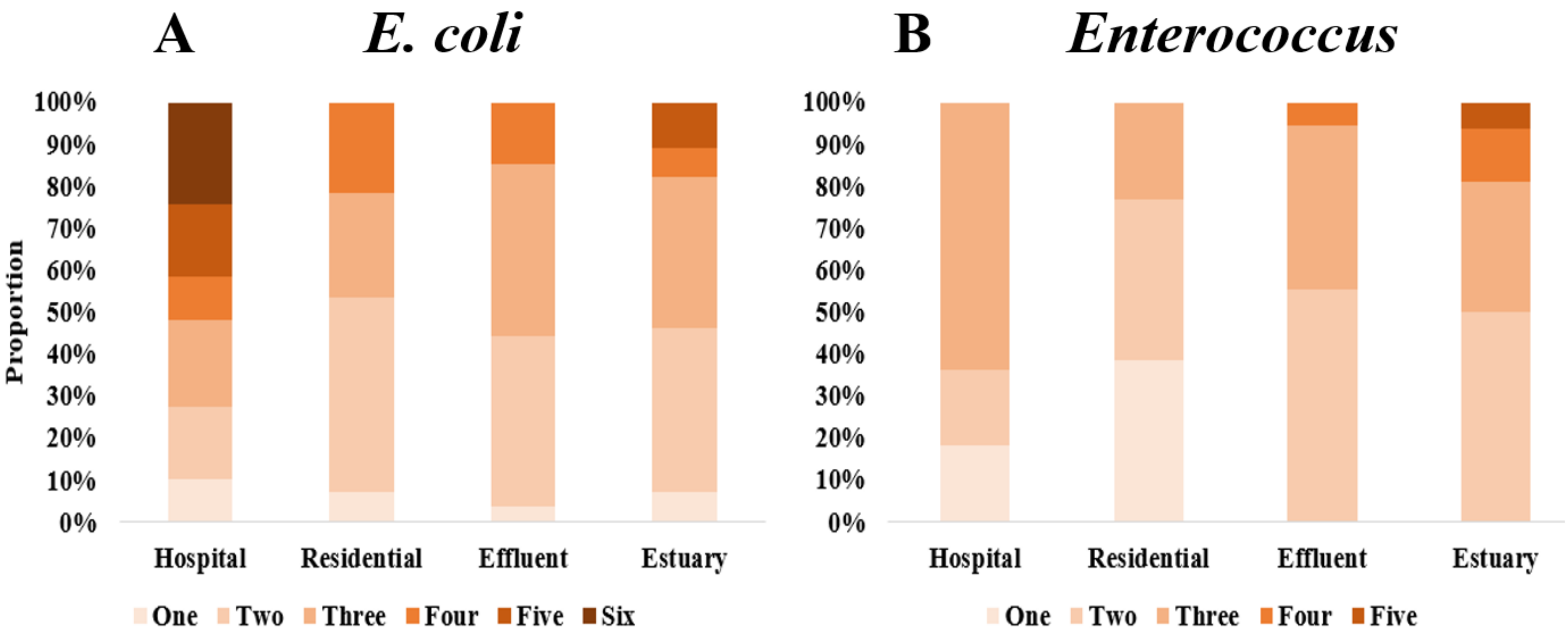


**Figure S3.** Proportion of isolates resistant to multiple classes of antibiotics among *E. coli* (panel A) and *Enterococcus* (panel B) isolates at each site. Sample size per site for *E. coli* and *Enterococcus* is as follows: Hospital influent (n = 27 and 11), residential influent (n=29 and 13), effluent (n = 28 and 18) and estuary (n=28 and 16). Color scale indicates number of resistance phenotypes: darker colors indicate more resistance phenotypes. The height of each block within a column indicates the proportion of isolates with a given number of resistance phenotypes.

**Table S1.** Statistical results for testing for differences in $log_{10}$ mean total and ampicillin-resistant *E. coli* and enterococci concentrations among sampling sites. Significant differences (p-values <0.05) are bolded. p-values associated with Tuckey HSD test are shown in the last column.

| FIB | Total | | Ampicillin-resistant | |
|---|---|---|---|---|
| | F statistic | P- value | F statistic | P- value |
| *E. coli* | 23.13 | **<0.001** | 9.4 | **0.002** |
| Enterococci | 7.01 | **0.006** | 0.5 | 0.687 |

**Table S2.** Significance of pairwise comparisons of $\log_{10}$ mean concentrations of ampR *E. coli* by site. Significant differences between sites (p-values <0.05) are bolded. No significant difference among $\log_{10}$ mean concentrations of ampicillin-resistant enterococci by site were observed, therefore a post-hoc test was not necessary.

| Pairwise comparison | *E. coli* |
|---|---|
| | Ampicillin-resistant |
| Residential-Hospital | **0.047** |
| Residential-Effluent | **0.014** |
| Residential-Estuary | **0.001** |
| Hospital-Effluent | 0.892 |
| Hospital-Estuary | 0.196 |
| Effluent-Estuary | 0.505 |

**Table S3.** Frequency of susceptibility of ampicillin-resistant *E. coli* isolates at each site to the following antibiotics: cefotaxime (CTX), ciprofloxacin (CIP), amoxicillin + clavulanate (AMC), gentamicin (GEN), tetracycline (TET), and trimethoprim sulfamethoxazole (SXT). Ampicillin is not shown as all isolates tested were fully resistant to ampicillin, the selective antibiotic in the isolation procedure.

| Antibiotic | Susceptible | Intermediate | Resistant |
|---|---|---|---|
| **AMC** | | | |
| Effluent | 0.85 | 0.11 | 0.04 |
| Estuary | 0.92 | 0.04 | 0.04 |
| Hospital | 0.86 | 0.10 | 0.04 |
| Residential | 0.79 | 0.21 | 0.00 |
| **CIP** | | | |
| Effluent | 0.96 | 0.00 | 0.04 |
| Estuary | 0.86 | 0.00 | 0.14 |
| Hospital | 0.55 | 0.04 | 0.41 |
| Residential | 0.89 | 0.00 | 0.11 |
| **CTX** | | | |
| Effluent | 0.78 | 0.15 | 0.07 |
| Estuary | 0.85 | 0.04 | 0.11 |
| Hospital | 0.52 | 0.00 | 0.48 |
| Residential | 0.79 | 0.04 | 0.18 |
| **GEN** | | | |
| Effluent | 0.81 | 0.04 | 0.15 |
| Estuary | 0.85 | 0.04 | 0.11 |
| Hospital | 0.52 | 0.00 | 0.48 |
| Residential | 0.93 | 0.00 | 0.07 |
| **SXT** | | | |
| Effluent | 0.44 | 0.00 | 0.56 |
| Estuary | 0.50 | 0.00 | 0.50 |
| Hospital | 0.24 | 0.00 | 0.76 |
| Residential | 0.46 | 0.00 | 0.54 |
| **TET** | | | |
| Effluent | 0.11 | 0.07 | 0.81 |
| Estuary | 0.14 | 0.00 | 0.86 |
| Hospital | 0.38 | 0.00 | 0.62 |
| Residential | 0.25 | 0.04 | 0.71 |

**Table S4.** Frequency of susceptibility of ampicillin-resistant *Enterococcus* isolates to the following antibiotics: ciprofloxacin (CIP), erythromycin (ERY), gentamycin (GEN), linezolid (LZD), tetracycline (TET), and vancomycin (VAN). Ampicillin is not shown as all isolates tested were fully resistant to ampicillin, the selective antibiotic in the isolation procedure.

| **Antibiotic** | **Susceptible** | **Intermediate** | **Resistant** |
|---|---|---|---|
| **CIP** | | | |
| Effluent | 0.17 | 0.55 | 0.28 |
| Estuary | 0.06 | 0.69 | 0.25 |
| Hospital | 0.27 | 0.27 | 0.46 |
| Residential | 0.84 | 0.08 | 0.08 |
| **ERY** | | | |
| Effluent | 0.28 | 0.39 | 0.33 |
| Estuary | 0.25 | 0.31 | 0.44 |
| Hospital | 0.46 | 0.27 | 0.27 |
| Residential | 0.54 | 0.23 | 0.23 |
| **GEN** | | | |
| Effluent | 0.88 | 0.06 | 0.06 |
| Estuary | 0.69 | 0.12 | 0.19 |
| Hospital | 1.00 | 0.00 | 0.00 |
| Residential | 0.93 | 0.07 | 0.00 |
| **LZD** | | | |
| Effluent | 0.94 | 0.06 | 0.00 |
| Estuary | 1.00 | 0.00 | 0.00 |
| Hospital | 1.00 | 0.00 | 0.00 |
| Residential | 1.00 | 0.00 | 0.00 |
| **TET** | | | |
| Effluent | 0.17 | 0.00 | 0.83 |
| Estuary | 0.12 | 0.00 | 0.88 |
| Hospital | 0.09 | 0.18 | 0.73 |
| Residential | 0.38 | 0.08 | 0.54 |
| **VAN** | | | |
| Effluent | 1.00 | 0.00 | 0.00 |
| Estuary | 1.00 | 0.00 | 0.00 |
| Hospital | 1.00 | 0.00 | 0.00 |
| Residential | 1.00 | 0.00 | 0.00 |

**Table S5.** Akaike's information criterion (AIC) values for logistic regression models applied to *E. coli* and *Enterococcus* datasets for susceptibility to additional antibiotics. AIC for the preferred model is in bold.

| **Model AIC** | ***E. coli*** | ***Enterococcus*** |
|---|---|---|
| Additive model (Site+Antibiotic) | 599.79 | **233.33** |
| Interaction model (Site*Antibiotic) | **593.47** | 243.96 |
| Interaction model with random effect (Site*Antibiotic + 1\|SampleID) | 958.70 | 479.90 |

**Table S6.** Influence of site and antibiotics on the log odds of *E. coli* resistance frequency to additional antibiotics. The model's intercept corresponds to site [residential wastewater], antibiotic [GEN] or their interaction [residential wastewater] * [GEN]. Significant relationships are bolded (p-values <0.05). The significant p-value for the intercept indicates that the predicted resistance is significantly different than 0.

| | **Resistance** | | |
|---|---|---|---|
| *Predictors* | *Log-Odds* | *CI* | *p* |
| (Intercept: Residential*GEN) | -2.56 *** | -4.39 – -1.36 | **<0.001** |
| Site [Hospital] | 2.50 ** | 1.06 – 4.43 | **0.002** |
| Site [Effluent] | 0.82 | -0.91 – 2.86 | 0.371 |
| Site [Estuary] | 0.44 | -1.43 – 2.53 | 0.641 |
| Antibiotic [TET] | 3.48 *** | 2.01 – 5.46 | **<0.001** |
| Antibiotic [CTX] | 1.04 | -0.60 – 3.05 | 0.240 |
| Antibiotic [SXT] | 2.71 ** | 1.27 – 4.65 | **0.001** |
| Antibiotic [CIP] | 0.44 | -1.43 – 2.53 | 0.641 |
| Site [Hospital] *Antibiotic [TET] | -2.92 ** | -5.11 – -1.08 | **0.003** |
| Site [Effluent] *Antibiotic [TET] | -0.25 | -2.61 – 1.91 | 0.823 |
| Site [Estuary] *Antibiotic [TET] | 0.43 | -2.00 – 2.75 | 0.714 |
| Site [Hospital] *Antibiotic [CTX] | -1.04 | -3.26 – 0.90 | 0.313 |
| Site [Effluent] *Antibiotic [CTX] | -1.82 | -4.54 – 0.58 | 0.153 |
| Site [Estuary] *Antibiotic [CTX] | -1.04 | -3.62 – 1.36 | 0.401 |
| Site [Hospital] *Antibiotic [SXT] | -1.49 | -3.69 – 0.37 | 0.137 |
| Site [Effluent] *Antibiotic [SXT] | -0.74 | -3.00 – 1.29 | 0.488 |
| Site [Estuary] *Antibiotic [SXT] | -0.59 | -2.90 – 1.55 | 0.591 |
| Site [Hospital] *Antibiotic [CIP] | -0.72 | -3.03 – 1.41 | 0.507 |
| Site [Effluent] *Antibiotic [CIP] | -1.95 | -5.42 – 0.80 | 0.192 |
| Site [Estuary] *Antibiotic [CIP] | -0.12 | -2.69 – 2.37 | 0.926 |
| Observations | 560 | | |
| $R^2$ Tjur | 0.314 | | |

* p<0.05   ** p<0.01   *** p<0.001

**Table S7.** Post-hoc comparisons of the logistic model on influence of site and antibiotics on the log odds of *E. coli* resistance frequency to additional antibiotics by (A) site and (B) antibiotic. Significant relationships are bolded (p-values <0.05), marginally significant relationships (p-values = 0.05) are italicized.

**(A)**

| Site | Estimate | Std. Error | z value | Pr(>\|z\|) |
|---|---|---|---|---|
| Hospital - Residential | 2.4960 | 0.8225 | 3.034 | **0.013** |
| Effluent - Residential | 0.8157 | 0.8157 | 0.894 | 0.804 |
| Estuary - Residential | 0.4447 | 0.9549 | 0.466 | 0.966 |
| Effluent - Hospital | -1.6802 | 0.6569 | -2.558 | **0.050** |
| Estuary - Hospital | -2.0513 | 0.7151 | -2.868 | **0.021** |
| Estuary - Effluent | -0.3711 | 0.8166 | -0.454 | 0.968 |

**(B)**

| Antibiotic | Estimate | Std. Error | z value | Pr(>\|z\|) |
|---|---|---|---|---|
| CTX - GEN | 1.0389 | 0.8843 | 1.175 | 0.760 |
| SXT - GEN | 2.7081 | 0.8259 | 3.279 | **0.009** |
| CIP - GEN | 0.4447 | 0.9549 | 0.466 | 0.990 |
| TET - GEN | 3.4812 | 0.8447 | 4.121 | **< 0.001** |
| SXT - CTX | 1.6692 | 0.6221 | 2.683 | *0.054* |
| CIP - CTX | -0.5942 | 0.7854 | -0.757 | 0.941 |
| TET - CTX | 2.4423 | 0.6469 | 3.775 | **0.002** |
| CIP - SXT | -2.2634 | 0.719 | -3.148 | **0.014** |
| TET - SXT | 0.7732 | 0.5644 | 1.370 | 0.640 |

**Table S8.** Influence of site and antibiotic on the log odds of *Enterococcus* resistance frequency to additional antibiotics. The model's intercept corresponds to site [residential wastewater] and antibiotic [GEN]. Significant relationships are bolded (p-values <0.05). The significant p-value for the intercept indicates that the predicted resistance is significantly different than 0.

| | **Resistance** | | |
|---|---|---|---|
| *Predictors* | *Log Odds* | *CI* | *p* |
| (Intercept: Residential+GEN) | -3.68*** | -5.12 – -2.48 | **<0.001** |
| Site [Hospital] | 1.06 | -0.00 – 2.17 | 0.052 |
| Site [Effluent] | 1.13* | 0.18 – 2.15 | **0.022** |
| Site [Estuary] | 1.50** | 0.53 – 2.54 | **0.002** |
| Antibiotic [TET] | 3.93*** | 2.81 – 5.29 | **<0.001** |
| Antibiotic [CIP] | 1.59 | 0.48 – 2.91 | **0.009** |
| Antibiotic [ERY] | 1.94** | 0.86 – 3.24 | **0.001** |
| Observations | 232 | | |
| $R^2$ Tjur | 0.310 | | |

* p<0.05 ** p<0.01 *** p<0.001

**Table S9.** Post-hoc comparisons of the logistic model on influence of site and antibiotics on the log odds of *Enterococcus* resistance frequency to additional antibiotics by (A) site and (B) antibiotic. Significant relationships are bolded (p-values <0.05).

**(A)**

| Site | Estimate | Std. Error | z value | Pr(>\|z\|) |
|---|---|---|---|---|
| Hospital - Residential | 1.06037 | 0.55176 | 1.922 | 0.207 |
| Effluent - Residential | 1.13013 | 0.49974 | 2.261 | 0.100 |
| Estuary - Residential | 1.50016 | 0.51083 | 2.937 | **0.009** |
| Effluent - Hospital | 0.06976 | 0.47453 | 0.147 | 0.999 |
| Estuary - Hospital | 0.43979 | 0.48133 | 0.914 | 0.680 |
| Estuary - Effluent | 0.37003 | 0.41909 | 0.883 | 0.681 |

**(B)**

| Antibiotic | Estimate | Std. Error | z value | Pr(>\|z\|) |
|---|---|---|---|---|
| TET - GEN | 3.9303 | 0.6207 | 6.332 | **< 0.001** |
| CIP - GEN | 1.5872 | 0.6048 | 2.624 | **0.0410** |
| ERY - GEN | 1.9369 | 0.5957 | 3.251 | **0.006** |
| CIP - TET | -2.343 | 0.4508 | -5.198 | **< 0.001** |
| ERY - TET | -1.9934 | 0.4362 | -4.57 | **< 0.001** |
| ERY - CIP | 0.3496 | 0.4196 | 0.833 | 0.834 |

**Table S10.** Antibiotic resistant profiles observed in *E. coli* isolates. Sample size (isolate number) per site is as follows: Hospital influent (n = 27), residential influent (n=29), effluent (n = 28) and estuary (n=28).

| | Site | | | | |
|---|---|---|---|---|---|
| **Combination** | **Hospital** | **Residential** | **Effluent** | **Estuary** | **Total** |
| AMP | 3 | 2 | 1 | 2 | 8 |
| AMP + GEN | 0 | 0 | 1 | 0 | 1 |
| AMP + CTX | 1 | 0 | 0 | 1 | 2 |
| AMP + TET | 2 | 10 | 9 | 9 | 30 |
| AMP + SXT | 2 | 3 | 1 | 1 | 7 |
| AMP + GEN + CTX | 1 | 0 | 0 | 0 | 1 |
| AMP + GEN + SXT | 0 | 0 | 1 | 0 | 1 |
| AMP + CTX + TET | 0 | 1 | 0 | 1 | 2 |
| AMP + CTX + SXT | 2 | 1 | 1 | 0 | 4 |
| AMP + TET + SXT | 3 | 5 | 9 | 9 | 26 |
| AMP + GEN + CTX + SXT | 2 | 2 | 0 | 0 | 4 |
| AMP + GEN + CIP + TET | 0 | 0 | 1 | 0 | 1 |
| AMP + CTX + AMC + TET | 0 | 0 | 0 | 1 | 1 |
| AMP + GEN + TET + STX | 0 | 0 | 1 | 0 | 1 |
| AMP + CIP + TET + SXT | 1 | 3 | 0 | 1 | 5 |
| AMP + CTX + TET + SXT | 0 | 1 | 1 | 0 | 2 |
| AMP + AMC + TET + SXT | 0 | 0 | 1 | 0 | 1 |
| AMP + GEN + CIP + TET + STX | 4 | 0 | 0 | 3 | 7 |
| AMP + CTX + AMC + TET + SXT | 1 | 0 | 0 | 0 | 1 |
| AMP + GEN + CIP + AMC + TET + STX | 7 | 0 | 0 | 0 | 7 |

**Table S11.** Antibiotic resistant profiles observed in *Enterococcus* spp. isolates. Sample size (isolate number) is as follows: Hospital influent (n = 11), residential influent (n= 13), effluent (n =18) and estuary (n=16).

| | Site | | | | |
|---|---|---|---|---|---|
| **Profile** | **Hospital** | **Residential** | **Effluent** | **Estuary** | **Total** |
| AMP | 2 | 5 | 0 | 0 | 7 |
| AMP + CIP | 1 | 0 | 0 | 0 | 1 |
| AMP + TET | 1 | 4 | 7 | 6 | 18 |
| AMP + ERY | 0 | 1 | 3 | 2 | 6 |
| AMP + CIP + TET | 4 | 1 | 3 | 2 | 10 |
| AMP + TET + ERY | 3 | 2 | 2 | 3 | 10 |
| AMP + GEN + CIP + TET | 0 | 0 | 0 | 1 | 1 |
| AMP + GEN + TET + ERY | 0 | 0 | 1 | 1 | 2 |
| AMP + GEN + CIP + TET + ERY | 0 | 0 | 0 | 1 | 1 |

**Table S12.** Antibiotics chosen for the Kirby-Bauer disc diffusion assay for *E. coli*.

| **Antibiotic** | **Class** | **Disk Content** |
|---|---|---|
| Ampicillin | Penicillins | 10 µg |
| Gentamycin | Aminoglycosides | 10 µg |
| Amoxicillin with clavulanate | Beta-Lactamase inhibitor combinations | 20/10 µg |
| Cefotaxime | Cephem | 30 µg |
| Ciprofloxacin | Fluoroquinolones | 5 µg |
| Trimethoprim-sulfamethoxazole | Folate pathway inhibitors | 1.25/23.75 µg |
| Tetracycline | Tetracyclines | 30 µg |

**Table S13.** Antibiotics chosen for the Kirby-Bauer disc diffusion assay for *Enterococcus* spp.

| **Antibiotic** | **Class** | **Disk Content** |
|---|---|---|
| Ampicillin | Penicillins | 10 μg |
| Gentamycin | Aminoglycosides | 120 μg |
| Linezolid | Oxazolidones | 30 μg |
| Vancomycin | Glycopeptides | 30 μg |
| Tetracycline | Tetracyclines | 30 μg |
| Erythromycin | Macrolides | 15 μg |
| Ciprofloxacin | Quinolones and Fluoroquinolones | 5 μg |
